# Supplementary material for: Genome-wide profiling of alternative splicing genes in hybrid poplar (P.alba×P.glandulosa cv.84K) leaves
Source: PLoS One. 2020 Nov 18;15(11):e0241914. doi: 10.1371/journal.pone.0241914 (PMC7673502; doi:10.1371/journal.pone.0241914)
Supplement: S1 Fig — (DOCX) [file pone.0241914.s001.docx]

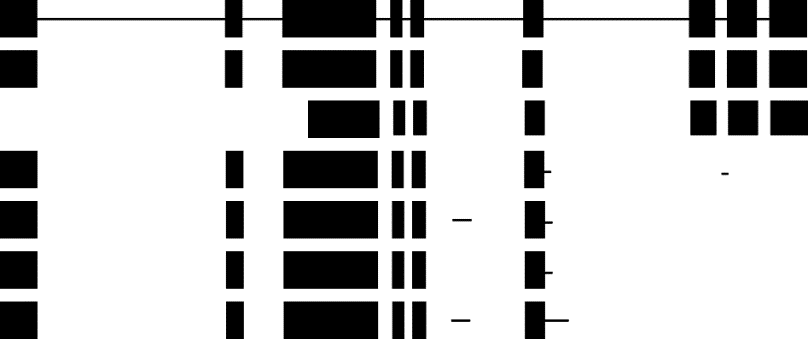


DNA

Isoform-1

Isoform-2

Isoform-3

Isoform-4

Isoform-5

Isoform-6

**S1 Fig. Transcripts of the gene Potri.002G124200.v3.0（PABP1）since of AS**
