# Supplementary material for: Genome-wide profiling of alternative splicing genes in hybrid poplar (P.alba×P.glandulosa cv.84K) leaves
Source: PLoS One. 2020 Nov 18;15(11):e0241914. doi: 10.1371/journal.pone.0241914 (PMC7673502; doi:10.1371/journal.pone.0241914)
Supplement: S2 Fig — The bases in frame indicated PCR primer. The isoform1 was normal transcript and isoform2 was included an intron. (DOCX) [file pone.0241914.s002.docx]

**
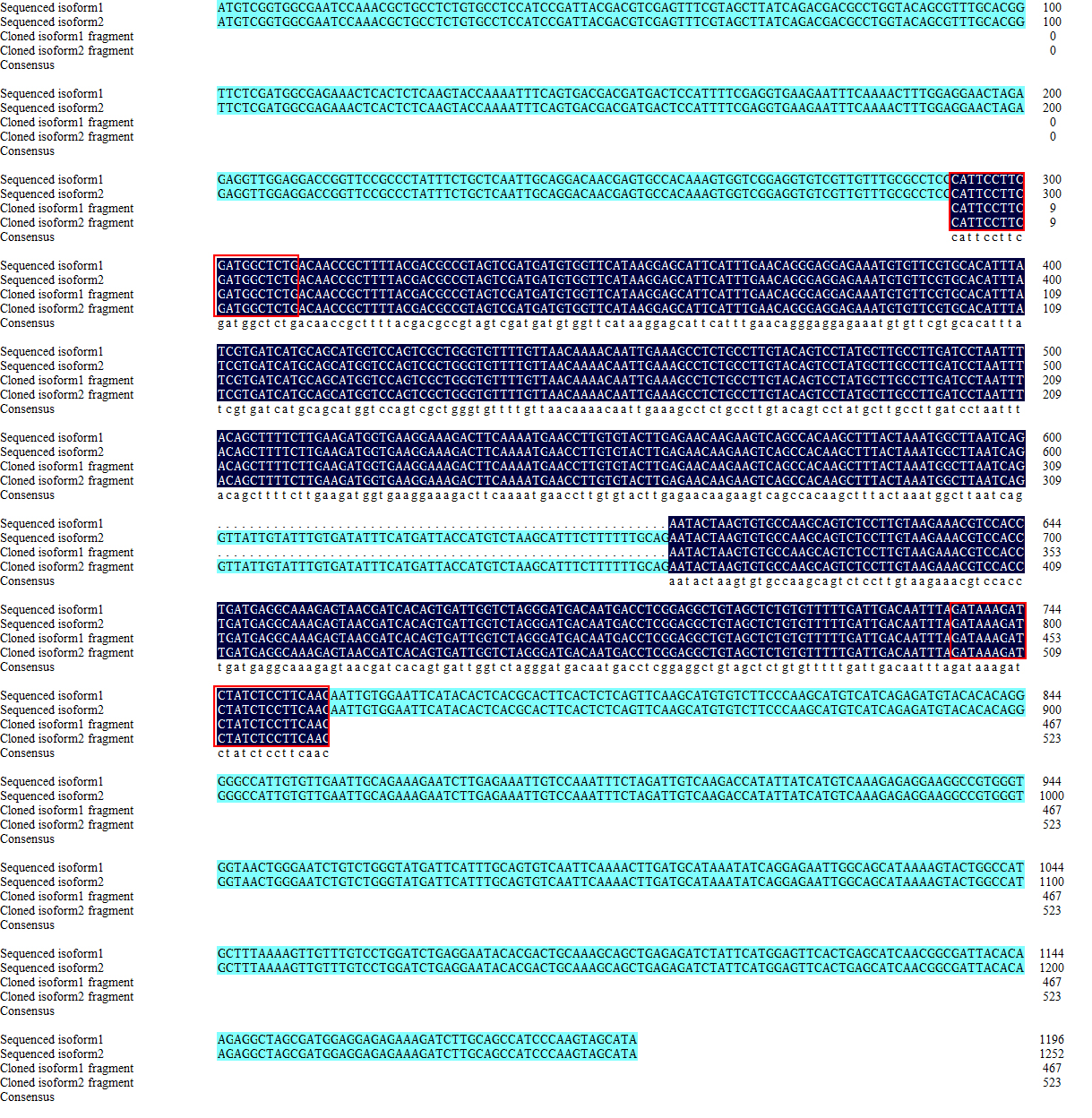
**

**S2 Fig. Transcripts alignment of two Illumina sequencing isoforms and two PCR fragments of the gene 6853.**

The bases in frame indicated PCR primer. The isoform1 was normal transcript and isoform2 was included a intron.
