# Supplementary figures and images for: Genome-wide profiling of alternative splicing genes in hybrid poplar (P.alba×P.glandulosa cv.84K) leaves
Source: PLoS One. 2020 Nov 18;15(11):e0241914. doi: 10.1371/journal.pone.0241914 (PMC7673502; doi:10.1371/journal.pone.0241914)

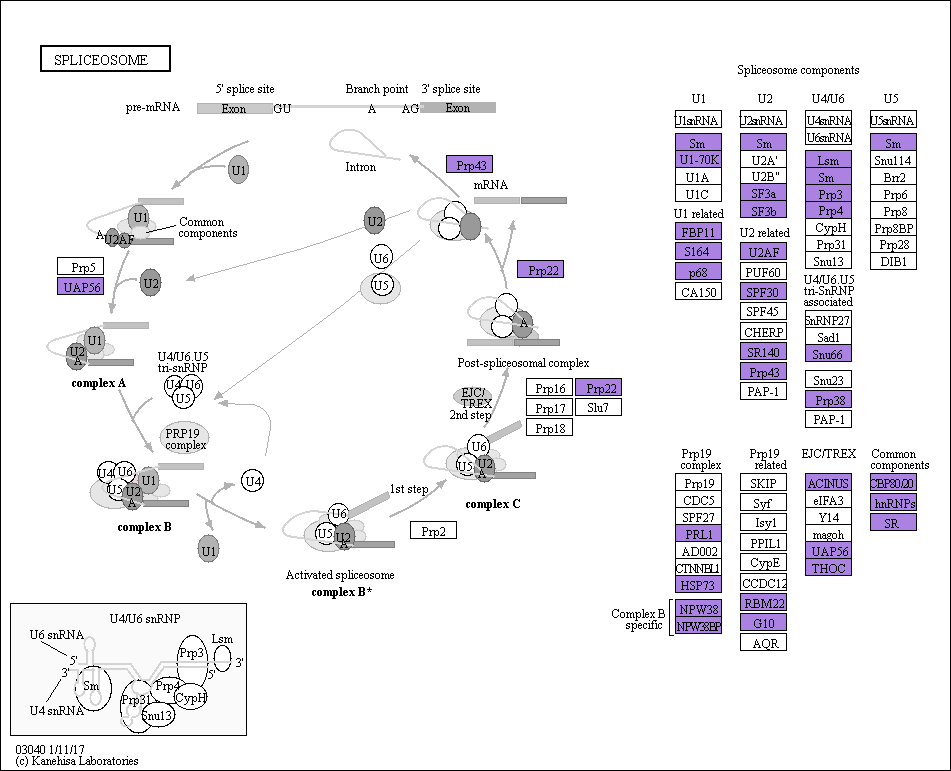


**S3 Fig. Splicesome pathway and AS genes distribution.**

The purple box represents AS genes.

Supplement: S3 Fig — The purple box represents AS genes. (DOCX) [file pone.0241914.s003.docx]
