# Supplementary material for: Genome-wide profiling of alternative splicing genes in hybrid poplar (P.alba×P.glandulosa cv.84K) leaves
Source: PLoS One. 2020 Nov 18;15(11):e0241914. doi: 10.1371/journal.pone.0241914 (PMC7673502; doi:10.1371/journal.pone.0241914)
Supplement: S1 Table — (DOCX) [file pone.0241914.s006.docx]

**S1 Table. The primer information for PCR validation**

| Gene ID | Forward primer (5’-3’) | Reversed primer(5’-3’) |
| --- | --- | --- |
| Gene1775 | ATGGCTAGGATTCAGGCAAAAC | CACATTGAGAGCGACTCC |
| Gene2340 | GCAAGCTTCTTTCTTCTTC | GCAGCTTCAAGATACTCTCTG |
| Gene14298 | CTACCTCCACAGTTTTTCGC | GAAGGCGCTTGATGAACGTG |
| Gene15649 | GGTGCTGATTCTCCCTCT | CTTCAACTCTCCGTAGGTC |
| Gene5375 | GCTCGTAACATGGCTCG | CTTCCAGGTCCTTCAGGAAC |
| Gene6853 | CATTCCTTCGATGGCTCTG | GTTGAAGGAGATAGATCTTTATC |
