# Supplementary material for: Genome-wide profiling of alternative splicing genes in hybrid poplar (P.alba×P.glandulosa cv.84K) leaves
Source: PLoS One. 2020 Nov 18;15(11):e0241914. doi: 10.1371/journal.pone.0241914 (PMC7673502; doi:10.1371/journal.pone.0241914)
Supplement: S2 Table — (DOCX) [file pone.0241914.s007.docx]

**S2 Table. Transcriptome sequenceing data of 84K poplar leaf**

| Catagory | Number or ratio |
| --- | --- |
| Clean reads | 23764540 |
| Clean bases | 7115797196 |
| GC Content | 44.93% |
| %≥Q30 | 94.98% |
| Mapped Reads | 16225148(68.27%) |
| Uniq Mapped Reads | 15669609（65.94%) |
| Multiple Map Reads | 555548(2.34%) |
