# Supplementary material for: Genome-wide profiling of alternative splicing genes in hybrid poplar (P.alba×P.glandulosa cv.84K) leaves
Source: PLoS One. 2020 Nov 18;15(11):e0241914. doi: 10.1371/journal.pone.0241914 (PMC7673502; doi:10.1371/journal.pone.0241914)
Supplement: S3 Table — (DOCX) [file pone.0241914.s008.docx]

**S3 Table. The top 10 enriched genes in each GO category**

| Category | GO.ID | Term | KS |
| --- | --- | --- | --- |
| Cellular component | GO:0005622 | intracellular | 7.00E-05 |
|  | GO:0005871 | kinesin complex | 0.00034 |
|  | GO:0044424 | intracellular part | 0.00037 |
|  | GO:0010282 | senescence-associated vacuole | 0.00156 |
|  | GO:0031981 | nuclear lumen | 0.00187 |
|  | GO:0005876 | spindle microtubule | 0.00201 |
|  | GO:0005794 | Golgi apparatus | 0.00296 |
|  | GO:0005634 | nucleus | 0.00378 |
|  | GO:1902494 | catalytic complex | 0.00425 |
|  | GO:0000439 | core TFIIH complex | 0.0059 |
|  | GO:0009543 | chloroplast thylakoid lumen | 0.00842 |
| Biological process | GO:0006396 | RNA processing | 7.60E-08 |
|  | GO:0006418 | tRNA aminoacylation for protein translation | 8.60E-06 |
|  | GO:0010114 | response to red light | 2.20E-05 |
|  | GO:0010218 | response to far red light | 3.20E-05 |
|  | GO:0007186 | G-protein coupled receptor signaling pathway | 3.40E-05 |
|  | GO:0006000 | fructose metabolic process | 6.40E-05 |
|  | GO:0035304 | regulation of protein dephosphorylation | 8.10E-05 |
|  | GO:0051276 | chromosome organization | 8.90E-05 |
|  | GO:0009791 | post-embryonic development | 9.50E-05 |
|  | GO:0001522 | pseudouridine synthesis | 0.00011 |
| Molecular function | GO:0050505 | hydroquinone glucosyltransferase activity | 1.20E-06 |
|  | GO:0005524 | ATP binding | 1.50E-06 |
|  | GO:0008569 | minus-end-directed microtubule motor activity | 5.30E-06 |
|  | GO:0008017 | microtubule binding | 5.40E-06 |
|  | GO:0009982 | pseudouridine synthase activity | 2.10E-05 |
|  | GO:0004812 | aminoacyl-tRNA ligase activity | 0.00023 |
|  | GO:0004439 | phosphatidylinositol-4,5-bisphosphate 5-phosphatase activity | 0.00028 |
|  | GO:0016811 | hydrolase activity, acting on carbon-nitrogen bonds | 0.00038 |
|  | GO:0004221 | ubiquitin thiolesterase activity | 0.00047 |
|  | GO:0004345 | glucose-6-phosphate dehydrogenase activity | 0.00051 |
