# Supplementary material for: Genome-wide profiling of alternative splicing genes in hybrid poplar (P.alba×P.glandulosa cv.84K) leaves
Source: PLoS One. 2020 Nov 18;15(11):e0241914. doi: 10.1371/journal.pone.0241914 (PMC7673502; doi:10.1371/journal.pone.0241914)
Supplement: Attachment — Submitted filename: Response to reviewers1.doc [file pone.0241914.s010.doc]

**Dear editor:**

Thanks for the Reviewers' comments. The reply for each point is as follows.

Reviewer #1: The revised manuscript have shown great improvement and authors have addressed most of the reviewers' comments. The manuscript is ready for publication if authors can address some minor comments as below:

1. Please be careful with typo and some of terms used as they are not accurate. Please make changes to these issues in your revised documents:
Line 141: " stand error" to "standard error"
Line 360: "...evaluated initially" to "...evaluated in this paper..."
Line 361: "...reading length..." to "..read length.."
Line 363: "...sequencing amounts.." to "...sequencing depth..."
Line 363: "...In next..." to "In future research, ,,,"
Line 364: "...whole length..." to "...full length isoform..."
Line 154: change to "...showed it is comprehensive..."
Line 378: sequencing alignment results
Reply: All were corrected

2. Line 141: which multiple comparison test is used?
Reply: Duncan's multiple range test was used in our experiment. The description was modified in the method section (Statistic analysis).

3. "...mRNA fragment randomization test, insert length test, and transcriptome sequencing data saturation test...". Any supporting table or figure for this?
Reply: The description was from the sequencing company but they did not provide the relative data. Thus, the sentence was removed.

4. The authors mentioned that the description of the objective has been modified to tailor to this study that focus on leaf as the major organ and to generate some initial data. However, I could not find the revised sentence where I expect to have both revision to the objective and justification of using leaf to be in both abstract and last paragraph of introduction. Also, please include the section, paragraph or line information in the authors' response to help the reviewer to identify the revision (at least for the major revision).
Reply: It was modified in line 13-14 in abstract, and last paragraph of introduction.

5. The conclusion in the abstract and main text body is not specifically linked to the objective of using leaf as sample for the AS study.

Reply: It was modified in line 23-25 in abstract, and main text body

Reviewer #2: All my comments have been addressed, only publication number 6 is not correctly named. Must be De Palma M et al.

Reply: It was corrected.
